# Primary adenocarcinoma of the rete testis: A case report and review of the literature

**DOI:** 10.3892/ol.2013.1708

**Published:** 2013-11-26

**Authors:** YE TIAN, WENQING YAO, LULU YANG, JIANZHONG WANG, ROMEL WAZIR, KUNJIE WANG

**Affiliations:** 1 Department of Urology, West China Hospital, Sichuan University, Chengdu, Sichuan 610041, P.R. China; 2 Department of Pathology, West China Hospital, Sichuan University, Chengdu, Sichuan 610041, P.R. China; 3 Department of Ultrasound, West China Hospital, Sichuan University, Chengdu, Sichuan 610041, P.R. China

**Keywords:** testicular neoplasms, adenocarcinoma, rete testis, ultrasonography

## Abstract

Primary adenocarcinoma of the rete testis is an extremely rare extratesticular neoplasm. Due to its low occurrence and the scarcity of data, sonographic characteristics of adenocarcinoma of the rete testis are still poorly defined. A 46-year-old male complained of swelling and pain in the right side of the scrotum. No associated symptoms were observed. Anti-tuberculosis chemotherapy yielded no response. Postoperative pathology revealed a diagnosis of poorly-differentiated adenocarcinoma of the rete testis. Using the ultrasonography features observed, combined with a review of current literature, the diagnosis and differential diagnosis of this neoplasm are presented.

## Introduction

Adenocarcinoma of the rete testis is a rare, highly aggressive tumor originating from the nonspermatogenic epithelium of the intratesticular excretory ducts. Only ~60 cases have been reported in the literature to date. Rete testis adenocarcinoma occurs most frequently in elderly males and is usually associated with a poor prognosis (1). The majority of cases present as a scrotal mass with diffuse enlargement of the testis. However, it is difficult to make a differential diagnosis with other testicular lesions, as rete testis adenocarcinoma also invariably presents with epididymitis, hydrocele, inflammatory lumps or inguinal hernia (2–4). The delayed diagnosis of right testis adenocarcinoma is often made by the pathologist following surgery, due to non-specific clinical presentation and symptoms. Ultrasound is a proven, safe diagnostic procedure with a high degree of sensitivity and specificity for testicular tumors (5). Due to the low prevalence of adenocarcinoma of the rete testis, the sonographic characteristics of this highly malignant tumor type have not been studied in sufficient depth. This report presents a case of primary adenocarcinoma of the rete testis, as confirmed pathologically. The diagnosis and differential diagnosis of this neoplasm, with regard to sonographic characteristics, are reviewed and discussed.

## Case report

### Patient presentation

A 46-year-old male was admitted with complaints of swelling and pain in the right side of the scrotum for a 1-year period. No associated symptoms were observed. Anti-tuberculosis chemotherapy was performed in Sichuan Provincial People’s Hospital (Chengdu, China), which yielded no response. Physical examination revealed a swelling of the right scrotum and hard nodules at the head of the right epididymis exhibiting severe tenderness. Written informed consent was obtained from the patient.

### Ultrasonography

**Figure 1:**
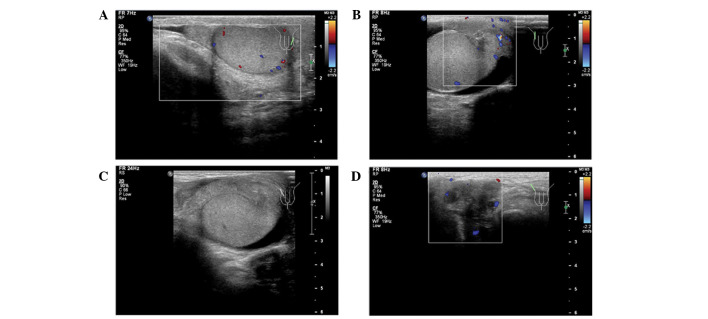
Sonography results revealed (A) normal sonographic characteristics in the left testis, (B) a hypoechoic mass at the right epididymis with a poorly-defined border. In addition, (C) color Doppler detected a relatively abundant blood flow in the mass and hydrocele and (D), a low mixed echo structure mass was localized in the right inguinal region with increased vascularization.

Ultrasonography of the scrotum revealed a 1.0×2.1-cm hypoechoic nodule at the right epididymis with a poorly-defined border. In addition, a relatively abundant blood flow was detected by color Doppler ultrasound and a weak echo was recorded in the right side of the tunica vaginalis area (Fig. 1B and C). A 5.0×1.9-cm irregular mass with an unclear boundary and low mixed echo structure was localized in the right inguinal region, which also showed increased vascularization (Fig. 1D).

### Diagnosis

The levels of lactate dehydrogenase (LDH), α-fetoprotein (AFP) and β-human chorionic gonadotropin (β-HCG) were normal in the serum. Chest radiographs, abdominal ultrasonography and computed tomography (CT) of the abdomen revealed no remarkable results. Considering the aforementioned results, a diagnosis of primary testicular tumor was proposed.

### Surgical procedures

Based on this provisional diagnosis, scrotal incision was performed for testicular exploration. This revealed blood-mixed fluid in the scrotal skin and an enlarged right epididymis with multiple yellow-grey, rough nodules, forming adhesions with the scrotal skin. Intraoperative frozen sections revealed adenocarcinoma in the specimen and the scrotal incision was extended and a high right inguinal orchidectomy was performed.

### Microscopy results

**Figure 2:**
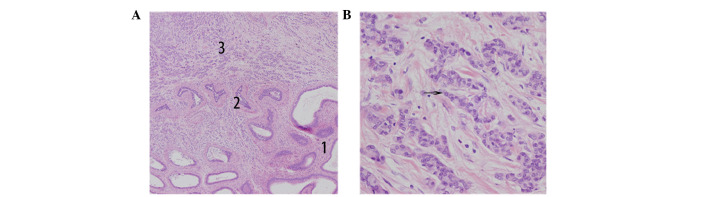
Light microscopic examination observations. (A) Histological transition from normal to tumor epithelium (magnification, ×40): 1, normal epithelium; 2, transitional epithelium; and 3, tumor epithelium. (B) Nests of cells: heterotypic hyperplasia and a glandular arrangement, as indicated by arrowhead (magnification, ×400).

Light microscopic examination revealed an absence of normal testicular microstructure, with nests of cells separated by fibrovascular stroma. In addition, a transition from normal to tumorous epithelium was detected. A small portion of lumens of the rete testis were packed with cuboidal tumorous cells. Cells exhibited heterotypic hyperplasia and were partially arranged in a glandular pattern, with hyperchromatic nuclei, an increase in the nuclear/cytoplasmic ratio and a visible nucleolus (Fig. 2). No involvement of the tumor to the testicular parenchyma or tunica was detected. A diagnosis of poorly-differentiated adenocarcinoma of the rete testis was established based on the diagnostic criteria of Nochomovitz and Orenstein (1).

### Patient outcome

Taking into account that adenocarcinoma of the rete testis is highly resistant to adjuvant radiotherapy and any known chemotherapeutic regimens (4,6), no further treatment was administered. Follow-up was carried out by serum LDH, AFP and β-HCG testing and abdominal CT every three months. The patient subsequently underwent metastasis at multiple sites and succumbed to adenocarcinoma 11 months following surgery.

## Discussion

Primary adenocarcinoma of the rete testis is resistant to adjuvant therapy and is associated with a poor prognosis. As many as 40% of patients succumb to this condition within one year of diagnosis. Survival rates for 3- and 5-years are 49 and 13%, respectively (4). Early diagnosis with surgical management is recommended by the majority of urologists (4,7–9). There are no specific clinical manifestations but tumor markers, including AFP and β-HCG, may help to detect the tumor earlier. CT-positron emission tomography may provide improved diagnostic sensitivity but is considered expensive and is not cost-effective (10). Ultrasound has been shown to be a reliable and valuable tool in the diagnosis of scrotal abnormalities. This procedure is relatively cheap and noninvasive. In addition, it provides real-time imaging, reveals internal blood flow properties, causes little discomfort and is easily repeatable, as well as being suitable for X-ray-sensitive organs as an ionizing radiation-free test. Ultrasound diagnostics are therefore recommended for confirming the presence of testicular masses (11,12).

Ultrasonography provides information regarding composition of the lesion which may facilitate diagnosis and differentiation from other pathological tumor types. Adenocarcinoma of the rete testis is typically located in the region of the epididymis or testicular hilum, rather than the intratesticular region, as reported in the majority of the current literature (3,8,13,14). The majority of patients exhibit hydrocele, and echoic paratesticular regions are observed (3,15,16). Nodular septations with cystic solid components have also been reported as an unusual observation which varies from other pathological tumor types (17). In the majority of cases, the lesions present as hypoechoic masses with poorly-defined borders, although an uneven echo pattern is detected on occasion (3,9). Increased vascularization of the tumor is another ultrasonographic feature which is helpful in differential diagnosis (8). The case presented in the current report is in accordance with the majority of these features.

In a number of cases, other imaging methods, including CT, magnetic resonance imaging and nuclear medicine, may be necessary to complete the imaging work-up of patients with testicular tumors. However, pathological examination is still the gold standard for diagnosis confirmation.

In conclusion, adenocarcinoma of the rete testis is an extremely rare tumor type with a poor prognosis. Sonography is the most promising tool for early diagnosis and increased case examples providing sonographic tumor observations must be presented to achieve an improved rate of diagnosis.
